# Typhoid Fever Surveillance and Vaccine Use — South-East Asia and Western Pacific Regions, 2009–2013

**Published:** 2014-10-03

**Authors:** Kashmira A. Date, Adwoa D. Bentsi-Enchill, Kimberley K. Fox, Nihal Abeysinghe, Eric D. Mintz, M. Imran Khan, Sushant Sahastrabuddhe, Terri B. Hyde

**Affiliations:** 1 Global Immunization Division, Center for Global Health, CDC; 2 Immunization, Vaccines, and Biologicals, World Health Organization; 3 Regional Office for the Western Pacific, World Health Organization; 4 Regional Office for South-East Asia, World Health Organization; 5 Division of Foodborne, Waterborne and Environmental Diseases, National Center for Emerging and Zoonotic Infectious Diseases, CDC; 6 Coalition Against Typhoid Secretariat, Sabin Vaccine Institute, Washington, DC; 7 International Vaccine Institute, Seoul, South Korea

Typhoid fever is a serious, systemic infection resulting in nearly 22 million cases and 216,500 deaths annually, primarily in Asia (1). Safe water, adequate sanitation, appropriate personal and food hygiene, and vaccination are the most effective strategies for prevention and control. In 2008, the World Health Organization (WHO) recommended use of available typhoid vaccines to control endemic disease and outbreaks and strengthening of typhoid surveillance to improve disease estimates and identify high-risk populations (e.g., persons without access to potable water and adequate sanitation). This report summarizes the status of typhoid surveillance and vaccination programs in the WHO South-East Asia (SEAR) and Western Pacific regions (WPR) during 2009–2013, after the revised WHO recommendations. Data were obtained from the WHO/United Nations Children’s Fund (UNICEF) Joint Reporting Form on Immunization, a supplemental survey of surveillance and immunization program managers, and published literature. During 2009–2013, 23 (48%) of 48 countries and areas of SEAR (11) and WPR (37) collected surveillance or notifiable disease data on typhoid cases, with most surveillance activities established before 2008. Nine (19%) countries reported implementation of typhoid vaccination programs or recommended vaccine use during 2009–2013. Despite the high incidence, typhoid surveillance is weak in these two regions, and vaccination efforts have been limited. Further progress toward typhoid fever prevention and control in SEAR and WPR will require country commitment and international support for enhanced surveillance, targeted use of existing vaccines and availability of newer vaccines integrated within routine immunization programs, and integration of vaccination with safe water, sanitation, and hygiene measures.

Typhoid fever is caused by the bacterium *Salmonella enterica* serovar Typhi (Typhi). Infection is transmitted via the fecal-oral route with most cases and deaths occurring among populations that lack access to safe drinking water and adequate sanitation and hygiene. The illness has nonspecific symptoms, making it difficult to distinguish clinically from other febrile illnesses (2) that might be endemic or cause epidemics in the same geographic areas, such as paratyphoid fever, dengue, and malaria. Severe systemic complications, including intestinal perforation and neurologic manifestations, have been well documented, and intestinal perforation is the most common cause of death from typhoid (3). Bacterial culture (of blood, bone marrow, or other sterile sites) is the gold standard for laboratory confirmation and antimicrobial susceptibility testing. Rapid antibody-based serologic tests are available (e.g., Widal test, Tubex TF, and TyphiDot), but are less sensitive and less specific than bacterial culture (4). Appropriate antibiotics shorten the duration of fever and bacterial shedding and reduce the case-fatality rate. However, resistance to available antibiotics is common, and the prevalence of resistance is increasing (3). Humans are the only reservoir for Typhi, and a long-term carrier state occurs.

Two safe and effective typhoid vaccines are licensed and marketed internationally, an injectable polysaccharide vaccine based on the purified Typhi Vi antigen (ViPS vaccine) for persons aged ≥2 years, and a live attenuated oral Ty21a vaccine available in capsule formulation for persons aged ≥5 years. One ViPS vaccine (Sanofi Pasteur) was prequalified by the World Health Organization (WHO) in 2011, enabling purchase by United Nations agencies; Gavi, the Vaccine Alliance (Gavi); and some international donors.* In 2008, WHO updated its position paper on typhoid vaccines and recommended programmatic use of the existing ViPS and Ty21a vaccines for endemic and epidemic disease control (Box). For this report, the status of typhoid surveillance and vaccine use in the 5-year period after the updated WHO recommendations was reviewed, focusing on SEAR and WPR, which had the highest estimated incidence rates at the time of the updated recommendations (1).

Information on typhoid surveillance during 2009–2013 was obtained from a supplemental survey of surveillance officers and from published reports. Data included information on type of surveillance, level at which surveillance is conducted (national versus subnational), age groups, case definitions, and laboratory confirmation. Typhoid vaccination information was obtained from the WHO/UNICEF Joint Reporting Form on Immunization data for 2009–2013, a survey of immunization program managers, and published literature. Data were collected on vaccines used, target populations (excluding travelers) and program strategies. Selected examples of large-scale typhoid vaccination programs also were reviewed. The information available varied in detail, and might not represent current and comprehensive data for all countries reviewed. Data on typhoid surveillance and vaccine use, respectively, were available from 30 (63%) and 31 (65%) of the 48 countries and areas of SEAR and WPR.

## Typhoid Surveillance Programs

Overall, 23 (48%) of 48 countries and areas of SEAR and WPR collected data on typhoid cases. Of these, 22 reported that typhoid was a notifiable disease, and 20 conducted surveillance activities, most through passive reporting at the national level (Table 1). Among the 14 countries that reported the year when surveillance started, almost all had existing systems before 2008. Six countries reported surveillance in selected sentinel sites (Table 1). Overall, 15 countries reported having standard case definitions, which varied widely by country. For example, case definitions included different durations of fever, ranging from “no duration specified” to “fever for at least 1 week.” Five of eight countries that provided case definitions included “bradycardia” (reduced heart rate), a relatively nonsensitive and nonspecific sign, for classifying a case as suspected or probable typhoid. Laboratory testing was reported by 19 countries; 17 countries reported conducting laboratory confirmation (blood culture [17 of 19], stool culture [15 of 19]), 10 countries reported use of Widal serologic testing, and one reported use of other rapid tests. Data regarding proficiency testing of the laboratories were unavailable. In India and Bangladesh, blood culture data on typhoid cases were available through invasive bacterial disease surveillance sites for pneumonia and meningitis.

## Typhoid Vaccination Programs

During 2009–2013, nine (19%) of 48 countries and areas in SEAR and WPR implemented a typhoid vaccination program or recommended vaccine use (excluding vaccination of travelers) (Table 2). In most countries that reported a typhoid vaccination program, vaccination (using ViPS vaccine) was targeted toward high-risk groups and/or food handlers. In addition, 11 countries (Australia, Cambodia, Fiji, India, Indonesia, Nepal, New Zealand, Philippines, Singapore, Sri Lanka and Thailand) reported typhoid vaccine use (ViPS or Ty21a) in the private sector.

China, India, and Vietnam initiated public sector typhoid vaccination programs before 2008, targeting preschool or school-aged children in selected geographic areas (Table 2). Nepal implemented a school-based ViPS vaccine demonstration program in the Kathmandu Valley in 2011 (Table 2), and efforts are ongoing to expand the program to school-aged children and food handlers as recommended by Nepal’s National Committee for Immunization. In addition, a mass typhoid vaccination campaign using the ViPS vaccine was conducted in Fiji in cyclone-affected and high-risk areas in 2010; >64,000 ViPS doses were administered, covering 7% of the total Fiji population (5). Approximately 10,000 vaccine doses were used to respond to a concurrent outbreak.

BOX **World Health Organization (WHO) recommendations on typhoid vaccine use, 2008**

Countries should consider the programmatic use of typhoid vaccines for controlling endemic disease.

- In most countries, only targeted vaccination of high-risk groups and populations will be required.
- Where appropriate, vaccine use should be harmonized with routine immunization programs.
- Immunization of preschool and school-aged children is recommended in areas where typhoid is a significant public health problem in these age groups.

Given the epidemic potential, typhoid vaccination is recommended for outbreak control.

Decisions regarding programmatic use should be based on a detailed knowledge of the local epidemiologic situation and other local factors, such as school enrollment rates, sensitivity of prevailing strains to relevant antimicrobials, and cost-effectiveness analyses.

Priority should be given to strengthening surveillance systems for typhoid fever, including sentinel-site surveillance for preschool and school-aged children.

Typhoid vaccination programs should be implemented in the context of other control efforts.

- Health education and health promotion.
- Training of health professionals in diagnosis and treatment.
- Improvements in water quality and sanitation.

**Source:** WHO position paper on typhoid vaccines (2008).

### Discussion

Despite the substantial and recognized disease burden (1), progress in typhoid disease surveillance and use of typhoid vaccine in SEAR and WPR has been limited during the 5 years since revision of the WHO recommendations for typhoid vaccines in 2008. Most countries had passive reporting systems, primarily through existing surveillance programs established before 2008, and culture-based surveillance was conducted in fewer than half of countries. Similarly, despite the establishment of typhoid vaccination programs in some countries in SEAR and WPR before 2008, only two instances of large-scale typhoid vaccination were noted since 2008.

Establishing and strengthening typhoid surveillance remains a challenge, and subnational variations in typhoid incidence are common. Among countries for which data were available, the majority reported having typhoid surveillance as part of the national notifiable disease surveillance system, although most often typhoid was included as part of passive reporting of acute febrile illnesses or general infectious diseases. Culture confirmation of suspected and probable cases continues to be limited. Although most countries reported using a standard case definition, the case definitions used varied widely. Available serologic tests, including the Widal test, have limited value because of poor sensitivity and specificity for typhoid diagnosis, and difficulty with standardizing reagents and interpreting values across different settings. Given the challenges in the clinical diagnosis of typhoid fever, updated surveillance standards and guidelines, including standard case definitions and quality assurance and quality control protocols for laboratories, need to be widely disseminated and their use encouraged. Culture confirmation remains the gold standard for typhoid diagnosis; laboratory capacity building (including proficiency testing for quality assurance and quality control) is needed to increase the accuracy of disease reporting and to facilitate monitoring of antimicrobial resistance, which is a growing problem.

During 2001–2003, the Diseases of the Most Impoverished Program conducted systematic population-based surveillance across five Asian countries (6). The disease burden data and a series of typhoid vaccine studies (7) were instrumental in guiding global policy recommendations for vaccine use. More recent high-quality epidemiologic data with culture confirmation and data on risk factors from multiple settings will help guide prevention and control activities in Asia. Opportunities need to be explored to include typhoid in existing laboratory-based surveillance systems with culture confirmation (e.g., invasive bacterial disease networks). Furthermore, newer disease burden estimates (8) that account for disease risk and accumulating evidence from other regions such as sub-Saharan Africa (9) also warrant an updated, global review of typhoid surveillance and vaccination programs.

Despite experience with large scale typhoid vaccination studies and successful implementation of programs, vaccine adoption since the revised WHO recommendations was limited in SEAR and WPR. In China and Vietnam, two countries with large-scale typhoid vaccination programs, typhoid incidence was reported to have declined steadily since vaccine use was initiated; improvements to water and sanitation infrastructure also were reported in Vietnam during this time (10). In Fiji, an evaluation of the disaster-response campaign showed that vaccination was feasible and played a role in reducing typhoid incidence in the vaccinated areas compared with pre-cyclone years (5).

What is already known on this topic?

Typhoid fever is an acute, systemic infection that represents an important cause of morbidity and mortality in the developing world with nearly 22 million cases and 216,500 deaths annually worldwide. Safe drinking water, adequate sanitation, appropriate personal and food hygiene, and typhoid vaccination are the most effective prevention and control strategies.

What is added by this report?

During the 5-year period after revision of the World Health Organization recommendations for typhoid vaccines in 2008, progress in typhoid surveillance and vaccine use has been limited in the South-East Asia and Western Pacific regions. During 2009–2013, surveillance or notifiable disease data on typhoid cases were collected in 23 (48%) of 48 countries and areas, and typhoid vaccination or recommendation for use was reported by nine (19%) of 48 countries and areas in these two regions.

What are the implications for public health practice?

Despite the substantial and recognized disease burden, typhoid fever remains a neglected disease in both the South-East Asia and Western Pacific regions. Coordinated action involving key stakeholders and partners at the regional and national levels is needed to create appropriate typhoid fever prevention and control policies and strategies, especially in settings with high incidence of disease.

Although the reasons for low typhoid vaccine use are not fully documented, multiple factors might have contributed. Countries might require data to ascertain local disease burden and to identify high-risk populations, for whom the recommended vaccination strategies apply, and lack of such data might be an impediment to justify vaccination programs. As countries introduce multiple new vaccines in their national immunization programs, typhoid vaccination might be a lower priority or lack adequate national or donor funding. Vaccine supply might be another potential barrier. For example, in 2012, Sanofi Pasteur recalled certain lots of the ViPS vaccine, which remains the only typhoid vaccine prequalified by WHO. An assessment of vaccine supply from both international and domestic manufacturers in multiple countries and country level policies regarding licensure and use, could help to elucidate supply and use constraints. Evaluation of typhoid vaccine impact in a variety of epidemiologic and programmatic contexts might contribute to the evidence to increase vaccine use.

Newer generation typhoid conjugate vaccines (TCVs) are under development, and when available, will be considered for funding support by Gavi. These vaccines are expected to have several advantages over ViPS and Ty21a vaccines, in particular, the potential to be immunogenic in children aged <2 years (facilitating incorporation in routine childhood immunization programs), to provide a booster effect (currently lacking for the ViPS vaccine), and a longer duration of protection. Two conjugate vaccines are licensed and being used in the private sector in India, and a third is undergoing licensure review in China. Seven additional TCV candidates are currently in different stages of preclinical and clinical development. Ongoing efforts aim to develop bivalent typhoid-paratyphoid vaccines to prevent enteric fever as a whole.

WHO recently convened a group of experts to review the available clinical data on TCVs.† It is anticipated that through well-designed research and postlicensure studies, additional data supporting the use of TCV in public health vaccination programs will be available in the next few years. In the meantime, WHO continues to recommend use of the licensed ViPS and Ty21a vaccines. TCV remains in Gavi’s investment strategy for potential future funding support when a WHO-prequalified conjugate vaccine becomes available. In addition to global policies, coordinated action involving key stakeholders and partners at the regional and national levels is needed. Review of existing data, establishment of high quality culture-based typhoid fever surveillance at selected sentinel sites, targeted use of existing or newer typhoid vaccines (with evaluation of their impact), and guidance for diagnosis and management of patients will be crucial toward building the evidence for appropriate typhoid prevention and control policies and strategies, especially for settings with high incidence of typhoid fever.

## Figures and Tables

**TABLE 1:** Characteristics of typhoid fever surveillance programs, by country or area* — WHO South-East Asia and Western Pacific regions, 2009–2013

Country or area	Type of program	Age groups under surveillance	Typhoid fever as a notifiable disease	Standard case definition in use	Laboratory confirmation of cases	Part of the Health Management Information system or integrated disease surveillance systems
**South-East Asia Region**						
Bangladesh	Details of national surveillance not available; surveillance data available through invasive bacterial disease surveillance†	Not available	Not available	Not available	Not available	Not available
Bhutan	Passive national reporting	NA	Yes	NA	NA	Yes
India	Passive national reporting as part of integrated disease surveillance program; additional surveillance at subnational levels in selected sites; surveillance data available through invasive bacterial disease surveillance§	All ages	Yes	Yes	Yes	Yes
Indonesia	Passive national reporting; additional reporting of suspected cases through an early warning system implemented in 24 provinces	All ages	Yes	No	Yes	Yes
Nepal	Passive national reporting; sentinel site surveillance (two sites)	All ages	Yes¶	No	Yes	Yes
Sri Lanka	Passive national reporting; sentinel site surveillance (six sites)	All ages	Yes	Yes	Yes	Yes
Thailand	Passive national reporting integrated with general infectious disease/vaccine preventable disease surveillance	All ages	Yes	Yes	Yes	Yes
**Western Pacific Region**						
Australia	Passive national reporting	All ages	Yes¶	Yes	Yes	Yes
Brunei	Passive national reporting	All ages	Yes¶	No	Yes	Yes
Cambodia	No systematic surveillance	NA	Yes	NA	NA	Yes
China	Passive national reporting; sentinel site surveillance in seven high-risk provinces (13 sites)	All ages	Yes¶	Yes	Yes	Yes
China, Hong Kong SAR	Passive reporting	All ages	Yes	Yes	Yes	No
Cook Islands	No systematic surveillance	NA	Yes¶	NA	NA	Yes
Fiji	Passive national reporting; additional national level laboratory-based surveillance system	All ages	Yes	Yes	Yes	Yes
Japan	Passive national reporting	All ages	Yes¶	Yes	Yes	Yes
Laos	Passive national reporting	All ages	Yes	Yes	Yes	No
New Zealand	Passive national reporting	All ages	Yes	Yes	Yes	Yes
Palau	Passive national reporting	All ages	Yes	Yes	Yes	Yes
Papua New Guinea	No systematic surveillance	NA	Yes	NA	NA	Yes
Philippines	Passive national reporting	All ages	Yes	Yes	Yes	Yes
Samoa	Passive national reporting	All ages	Yes	Yes	Yes	Yes
Singapore	Passive national reporting	All ages	Yes¶	Yes	Yes	Yes
Vietnam	Passive national reporting; additional sentinel surveillance with laboratory confirmation of cases (3 sites)	All ages	Yes¶	Yes	Yes	Yes

**Abbreviations:** WHO = World Health Organization; NA = not applicable; SAR = Special Administrative Region.

* Countries or areas for whom data were available. The following countries and areas reported having no typhoid surveillance and typhoid as not being a notifiable disease: Kiribati, Nauru, Nuie, Solomon Islands, Timor Leste, Tokelau and Tuvalu.

† Additional information available at http://www.coalitionagainsttyphoid.org/wp-content/uploads/2014/09/05.Saha_.8TC.pdf.

§ Source: Pitzer VE, Bowles CC, Baker S, Kang G, Balaji V, Farrar JJ, et al. Predicting the impact of vaccination on the transmission dynamics of typhoid in South Asia: a mathematical modeling study. PLoS Negl Trop Dis 2014;8:e2642.

¶ System captures both typhoid fever and enteric fever overall.

**TABLE 2:** Summary of typhoid vaccination programs or recommended use (excluding vaccination of travelers), by country or area — WHO South-East Asia and Western Pacific regions, 2009–2013*

Country or area	National policy (year issued)	Targets for vaccination (excluding travelers)	Type of vaccine(s)
**South-East Asia Region**			
India	No	State of Delhi incorporated into the routine immunization program; since 2005, approimately 300,000 children aged 2–5 years vaccinated with a locally produced ViPS vaccine†	ViPS
Nepal	Yes (2012)	Subnational; school-aged children, food handlers In 2011, approximately 150,000 schoolchildren vaccinated with ViPS; estimated coverage of 65%§	ViPS
Sri Lanka	Yes (circa 1970)	National; food handlers, high-risk groups	ViPS
**Western Pacific Region** ¶			
Australia	Yes (2008)	National; military personnel, laboratory workers routinely working with Typhi	Ty21a and ViPS
Brunei	No	Food handlers	ViPS
China	No	Subnational; selected high-risk groups**	ViPS
South Korea	Not available	National; high-risk groups	ViPS
Malaysia	Not available	Subnational; food handlers	ViPS
Vietnam	Yes (1997)	Subnational (selected high-risk provinces); during 2000–2013, more than 5.6 million doses of domestically-produced ViPS vaccine administered to children aged 3–10 years in selected high-risk districts††	ViPS

**Abbreviations:** WHO = World Health Organization; ViPS = parenteral Vi polysaccharide; Ty21a = live, attenuated mutant strain of Typhi.

* The data presented reflect typhoid vaccination any time during the review period in countries or areas for whom data were available. The following countries and areas reported no typhoid vaccination in either public or private sector: Bhutan, Cook Islands, Japan, Kiribati, Nauru, Nuie, Palau, Papua New Guinea, Samoa, Solomon Islands, Timor Leste, Tokelau and Tuvalu.

† Additional information available at http://www.coalitionagainsttyphoid.org/wp-content/uploads/2014/09/12.DewanByOchiai.8TC.pdf.

§ Source: Sahastrabuddhe S, International Vaccine Institute; personal communication, August 2014.

¶ Mandatory vaccination of food handlers in Singapore (since the 1970s) was rescinded in 2010; therefore, Singapore is not included.

** Not used in national immunization program. Provinces choose their own strategies, including school-based vaccination of children in high-risk areas, vaccination of food handlers, outbreak-response vaccination, and vaccination for a wide age range in high-risk areas of high-risk provinces. Source: Control of typhoid fever through vaccination: China’s experience. Workshop report on review of typhoid fever vaccination programs in the People’s Republic of China, Guilin 2010. International Vaccine Institute 2010. Available at http://viva.ivi.int/ReportsandDocuments/Workshop%20report%20on%20review%20of%20typhoid%20fever%20vaccination%20programs%20in%20the%20People%27s%20Republic%20of%20China,%20Guilin%20Jun%202010.pdf.

†† Additional information available at http://www.coalitionagainsttyphoid.org/wp-content/uploads/2014/09/43.Cuong_.8TC.pdf.

## Footnotes

* Additional information available at http://www.who.int/immunization_standards/vaccine_quality/pq_system/en.

† Additional information available at http://www.who.int/immunization/research/meetings_workshops/typhoidvaccines_july14/en.
